# Supplementary material for: A guide to group effective connectivity analysis, part 1: First level analysis with DCM for fMRI
Source: Neuroimage. 2019 Oct 15;200:174–90. doi: 10.1016/j.neuroimage.2019.06.031 (PMC6711459; doi:10.1016/j.neuroimage.2019.06.031)
Supplement: Multimedia component 1 [file mmc1.docx]

**Supplementary text: Prior on time constants (intrinsic connections)***A guide to group effective connectivity analysis, part 1: first level analysis with DCM for fMRI*

Figure A.2 (middle and right) of the main text plot the prior time constants for DCM for fMRI. These were generated as follows. DCM estimates log scaling parameters $a_{I}$ and $b_{I}$ in place of the connectivity parameters $a$ and $b$. These have normal prior densities:

$$p\left( a_{I} \right)=N\left( \mu_{a},\sigma_{a}^{2} \right)$$

$$p\left( b_{I} \right)=N\left( \mu_{b}, \sigma_{b}^{2} \right)$$

$$\mu_{a}=\mu_{b}=0$$

$$\sigma_{a}^{2}=\frac{1}{64}$$

$$\sigma_{b}^{2}=1$$

The prior time constant can be expressed as a function of these log scaling parameters:

$$\tau\left( \mu_{a},\mu_{b} \right)=-\frac{1}{\text{pA}\cdot\exp\left( \mu_{a}+\mu_{b} \right)}$$

$$=-\frac{1}{pA}\cdot\exp\left( -\mu_{a}-\mu_{b} \right)$$

$$\text{pA}=-0.5\text{Hz}$$

This gives the prior on the time constant a lognormal distribution, which we write here in terms of its mean and variance:

$$\tau\left( \mu_{a},\mu_{b} \right) \sim\text{Lognormal}\left( \mu_{\tau}, \sigma_{\tau}^{2} \right)$$

$$\mu_{\tau}=\ln\tau\left( \mu_{a},\mu_{b} \right)$$

$$=\ln\left( -\frac{1}{pA} \right)-\mu_{a}-\mu_{b}$$

$$\sigma_{\tau}^{2}=\sigma_{a}^{2}+\sigma_{b}^{2}$$

The 90% credible interval is given by the lognormal inverse Cumulative Density Function (CDF) $\text{Φ}^{-1}$:

$$c=[\text{Φ}^{-1}\left( 0.05,\mu_{\tau}, \sigma_{\tau}^{2} \right)\text{ Φ}^{-1}(0.95,\mu_{\tau}, \sigma_{\tau}^{2})]$$
